# Impact of Phenylpropanoid Compounds on Heat Stress Tolerance in Carrot Cell Cultures

**DOI:** 10.3389/fpls.2016.01439

**Published:** 2016-09-22

**Authors:** Mauro Commisso, Ketti Toffali, Pamela Strazzer, Matteo Stocchero, Stefania Ceoldo, Barbara Baldan, Marisa Levi, Flavia Guzzo

**Affiliations:** ^1^Department of Biotechnology, University of VeronaVerona, Italy; ^2^S-IN Soluzioni InformaticheVicenza, Italy; ^3^Department of Biology, University of PaduaPadua, Italy

**Keywords:** anthocyanins, biological role of secondary metabolites, cultured cells, heat stress, hydroxycinnamic acids, phenylpropanoids, untargeted metabolomics

## Abstract

The phenylpropanoid and flavonoid families include thousands of specialized metabolites that influence a wide range of processes in plants, including seed dispersal, auxin transport, photoprotection, mechanical support and protection against insect herbivory. Such metabolites play a key role in the protection of plants against abiotic stress, in many cases through their well-known ability to inhibit the formation of reactive oxygen species (ROS). However, the precise role of specific phenylpropanoid and flavonoid molecules is unclear. We therefore investigated the role of specific anthocyanins (ACs) and other phenylpropanoids that accumulate in carrot cells cultivated *in vitro*, focusing on their supposed ability to protect cells from heat stress. First we characterized the effects of heat stress to identify quantifiable morphological traits as markers of heat stress susceptibility. We then fed the cultures with precursors to induce the targeted accumulation of specific compounds, and compared the impact of heat stress in these cultures and unfed controls. Data modeling based on projection to latent structures (PLS) regression revealed that metabolites containing coumaric or caffeic acid, including ACs, correlate with less heat damage. Further experiments suggested that one of the cellular targets damaged by heat stress and protected by these metabolites is the actin microfilament cytoskeleton.

## Introduction

Phenylpropanoids and flavonoids are two important classes of plant phenolic specialized metabolites widely distributed in plant kingdom. They are necessary for a wide range of processes, including seed dispersal, attraction of pollinators, pigmentation of fruits and flowers (Tanaka et al., 2008), regulation of auxin transport (Peer and Murphy, 2007), UV-B and photoprotection (Agati and Tattini, 2010), mechanical support (Frei, 2013), protection against insect herbivory and pathogens (Cohen et al., 2001; Lattanzio et al., 2006; War et al., 2012), pollen germination (Paupière et al., 2014) and communication with the rhizosphere (Weston and Mathesius, 2013).

Phenylpropanoids and flavonoids are believed to play a key role in the protection of plants against biotic and abiotic stress, in many cases by inhibiting the formation of ROS via a range of different mechanisms (Mierziak et al., 2014). This role may explain why diverse forms of biotic and abiotic stress result in the accumulation of one or more phenolic compounds (Dixon and Paiva, 1995; Jahangir et al., 2009). Although there is a large body of literature describing the relationships between stress and phenylpropanoid and/or flavonoid accumulation in plant cells and tissues, the precise role of individual molecules is unclear. The roles of individual metabolites is difficult to determine because plant cells accumulate a diverse spectrum of compounds, often by the modification of a smaller number of basic structures. In the phenylpropanoid and flavonoid families, the basic structures are often converted into glycosylated derivatives, esters with both aliphatic and aromatic organic acids, and amides with nitrogen-containing metabolites such as aminoacids and polyamines (Dixon and Paiva, 1995; Macoy et al., 2015). These decorations confer different physicochemical properties that have been investigated and partially elucidated *in vitro*. In the case of ACs, which is a sub-class of flavonoids, glycosylation results in redder pigmentation, aromatic acylation (usually with HBAs and HCAs) results in a blue shift and also greater molecular stability, and aliphatic acylation increases molecular stability and solubility (Tanaka et al., 2008). The implications of these different modifications *in vivo* is not well understood, and little is known about their involvement in specific biological functions.

Pigmented carrot (*Daucus carota* L.) cell lines provide a useful system to investigate the role of various phenylpropanoids and the biological relevance of AC modification and diversification. They accumulate a range of well-characterized ACs with a common cyanidin core structure. We have previously shown that carrot cell cultures respond to mechanical stress by accumulating more ACs, HCAs, and HBAs (all of which can scavenge ROS *in vivo*) thus protecting the cultures from ROS-induced cell death (Ceoldo et al., 2005, 2009). Carrot cell lines that accumulate ACs predominantly synthesize eight specific cyanidin derivatives, included some acylated with coumaric, caffeic, ferulic, and sinapic acids, described in detail by Gläßgen and Seitz (1992) and Ceoldo et al. (2009). These carrot cell lines also accumulate various coumaric, caffeic, ferulic, and sinapic acid derivatives (Ceoldo et al., 2009). We have also previously established a method to increase the synthesis of particular ACs by feeding cells with specific HCAs (*p*-coumaric, caffeic, ferulic, or sinapic acid) as acylation substrates (Toffali et al., 2013).

Since more extreme climatic conditions are expected in the future (Deryng et al., 2014), here we investigated the ability of individual ACs and HCAs to protect carrot cells from the deleterious effects of heat stress. We first characterized the effects of heat stress on cells to identify markers of heat stress susceptibility that can be screened by fluorescence microscopy, confocal microscopy and TEM, allowing the serial observation of cell cultures during heat stress and recovery. A specific quantitative marker of heat stress susceptibility was then used to determine the performance of cells under heat stress, comparing control cells with those fed on substrates that facilitate the accumulation of specific ACs and HCAs. Multivariate statistical analysis was then used to identify metabolites responsible for the prevention of heat damage.

## Materials and Methods

### Cell Culture

The carrot (*D. carota* L.) cell line R3M was grown and maintained as described by Toffali et al. (2013).

### Heat Stress and Cell Characterization

A 6-mL aliquot of 13-day-old R3M suspension culture was transferred to a Petri dish (6 cm diameter) and incubated at 44°C (20, 30, 40, 60, and 120 min) in a Memmert BE200 Bench Top Incubator in the dark since no light was present in the incubator. Each Petri dish was considered as a biological replicate and three replicates were used for each exposure time. Control samples (three biological replicates) were kept, for the same exposure times, at 25 ± 1°C in the dark. After the heat treatment, samples were maintained in the growth chamber for recovery. The recovery time points were established after preliminary serial microscopy observations of cell morphology over time. We then diluted 100 μL of stressed or control cells 1:10 with fresh growth medium, and cell viability and morphology were monitored by fluorescence microscopy after staining the cells with 5 μg/mL FDA (Sigma–Aldrich, St Louis, MO, USA) for recovery times of 3, 24, and 48 h. A stock solution of 1 mg/mL FDA in acetone was stored at 4°C in the dark, and diluted in milliQ water (1:10) just before use. Samples were observed after incubating for 5 min at room temperature in the dark. The proportions of viable and dead cells and those showing specific morphologies were assessed using a Nageotte counting chamber (height 0.5 mm) and counting ∼500 cells per biological replicate.

To follow heat stress effects over longer periods, cells prepared as described above were heat stressed at 44°C for 1 h or maintained at 25°C (controls). The fate of heat-stressed and control cells was followed using a Nageotte counting chamber following FDA staining as above, after 3, 24, 48, 72, 144, 216, 240, 312, 336, and 408 h recovery in the growth chamber. We added 2 mL of fresh growth medium to heat-stressed and control samples after 150 h recovery and ∼600 stressed and control cells were counted at each time point. The experiment was carried out twice.

Heat stress was also applied to cells fed with AC acylation substrates (*p-*coumaric, caffeic, ferulic, or sinapic acids). Targeted modification of the metabolome by feeding was carried out as described by Toffali et al. (2013). Briefly, 12-day-old R3M cell suspension cultures were fed with 0.4 mM HCA (caffeic acid, coumaric acid, ferulic acid, or sinapic acid). The HCAs were dispensed from 0.15 M stock solutions in methanol, except sinapic acid (0.1 M in methanol), and an equivalent amount of methanol was added to the controls. After feeding the cells for 4 h, the culture medium was discarded by centrifugation (220 × *g*, 5 min, 4°C) and replaced with an equivalent volume of conditioned medium from untreated 12-day-old R3M cell suspensions.

Control cells and fed cells were heat stressed for 1 h at 44°C, 24 h after feeding. The effects of heat stress on the control cells and fed cells were assessed 24 h after heat stress by determining the proportion of normal cells and cells with heat-induced damage (cytoplasmic patches) using a Nageotte chamber to count ∼1200 cells following FDA staining as above. To avoid unintentional bias, the operator was blinded to the contents of the Nageotte counting chamber. The cell counting was a time consuming task, so only one treated (fed) and one untreated (control) sample were included in each experiment, and the experiments were carried out six times for *p*-coumaric acid feeding, four times for ferulic and sinapic acid feeding, and twice for caffeic acid feeding.

Control cells and heat-stressed cells were also characterized by fluorescence and confocal microscopy using different fluorescent dyes. For ER staining, ER tracker^TM^ *blue-white* DPX powder was dissolved to a concentration of 1 mM in 50 μL DMSO. This stock solution was diluted 1:10 in DMSO (intermediate solution) and then added to the cells at a final concentration of 10 μM. Cells were kept in darkness for 15 min at room temperature. The stock and intermediate solutions were stored at -20°C. For mitochondrial staining, 1 mM of Mitotracker^®^Green FM stock solution in DMSO was diluted to 75 μM in methanol and stored at -20°C. Stressed and control cells were stained with a final concentration of 500 nM and observed after 45 min at room temperature in the dark. Membranes and lipid droplets were stained by diluting Nile Red 100 μg/mL stock solution in acetone 1:100 with water and adding the solution to the stressed and control cells at a final concentration of 1 μg/mL. Acid compartments were stained with LysoTracker^®^ Red DND-99 (Molecular Probes) at final concentrations of 75, 100, 150, 200, 250, 500, and 1000 nM. The cells were incubated for 30, 60, or 120 min at room temperature in darkness, washed with Gamborg B5 medium and at least 100 cells were immediately observed at each stain concentration. To highlight vesicle movement, a FM^®^1–43 stock solution of 5 mg/mL in water (stored at -20°C) was added to cells at a final concentration of 5 μg/mL. Cells were observed using a Leica DMRB fluorescence microscope with the following filter combinations: band pass filter 470–490 nm, dichroic mirror 510 nm, long pass filter >520 nm for FDA, Mitotracker^®^Green and Nile Red staining; band pass filter 340–380 nm, dichroic mirror 400 nm, long pass filter >430 nm for ER tracker^TM^ *blue-white* DPX staining.

For confocal microscopy, control cells and stressed cells 24 and 48 h after heat stress were double-stained with 10 μM ER tracker^TM^ *blue-white* DPX and 5 μg/mL FDA. ER tracker^TM^ *blue-white* DPX was added first and FDA was added 10 min later. Samples were maintained in darkness at room temperature for a further 15 min and then observed under a confocal microscope (TCS-SP5, Leica Microsystems, Wetzlar, Germany) by setting the 405 diode at 15% power intensity, and ER tracker^TM^ *blue-white* DPX fluorescence was recorded from 435 and 475 nm. The argon laser was set at 30% power intensity and the 488 nm laser line was set at 30% power intensity. FDA fluorescence was recorded at 500–570 nm. To detect ACs in the cytoplasm, red autofluorescence was recorded at 680–740 nm, with an excitation wavelength of 543 nm. The final images (1024 pixels × 1024 pixels) were processed using ImageJ software^1^ to determine the number of red pixels in the cytoplasm and vacuole.

### Colchicine and Cytochalasin D Treatments

We transferred 2-mL aliquots of the 13-day-old R3M suspension cell culture into Petri dishes (2 cm diameter). Colchicine powder (Sigma–Aldrich) was dissolved in DMSO to a concentration of 10 mM and was added to the cells at final concentrations of 0.08, 0.5, 1 (Lloyd et al., 1980; Thion et al., 1996) and 3 mM. The effects were monitored by confocal and/or fluorescence microscopy after 1, 5, 24, 48, 72, and 144 h by FDA staining as above. Control samples were treated with an equivalent volume of DMSO. Each experiment was carried out at least three times.

Cytochalasin D (Sigma–Aldrich) as a 5 mg/mL stock in DMSO was initially diluted to an intermediate concentration of 0.1 mg/mL in DMSO and added to the R3M cells at final concentrations of 0.02, 0.05, and 0.2 mM. The doses were based on previous experiments using 0.01 mM (Lloyd and Traas, 1988) and 0.02 mM (Traas et al., 1987). FDA-stained cells were monitored after 1, 5, 24, 48, 72, and 96 h by confocal and/or fluorescence microscopy. Equivalent volumes of DMSO were added to the control samples. Each experiment was carried out at least three times.

### Glucose/Glucose Oxidase Treatment

The oxidative stress induction was performed as previously described (Zottini et al., 2002; Ceoldo et al., 2005).

### *In vivo*-Imaging

Cells treated at 44°C for 1 h as described above were spread over a thin layer of solid growth medium (containing 0.7% agar) in a Petri dish (6 cm diameter). The cells were observed under an inverted Olympus IX70 microscope equipped with a JVC KY F58 3-CCD camera, and the images were acquired using Image Pro-Plus software (Media Cybernetics, Silverspring, MD, USA). A microscope field was chosen containing approximately 50 cells at low magnification. Higher magnification phase contrast images were acquired 24 h after the beginning of the experiment in order to check for the presence/absence of heat-induced cytoplasmic patches. Cells were monitored for 6 days, automatically recording a photogram every 20 min.

### Transmission Electron Microscopy (TEM)

Preparation of R3M cells for TEM experiments were carried out as described by Zuppini et al. (2007). The cell ultrastructure was observed using a Tecnai 12-BT transmission electron microscope (FEI, Eindhoven, the Netherlands) operating at 120 kV equipped with a Tietz camera.

### PIP Treatment

A concentrated PIP stock solution was diluted in DMSO and added at the final concentration of 0.1 mM to Petri dishes containing 6 mL of a 10-day-old R3M culture. Non-treated control cells were administered with an equivalent volume of DMSO. After incubation for 24 h, the cells were gently centrifuged (220 × *g*, 5 min, 4°C) and the supernatant was replaced with 11-day-old conditioned medium from another batch of cells in order to remove the residual PIP while avoiding any effects caused by fresh medium. After 4 h without PIP, the cells were heat stressed at 44°C for 1 h and the morphology of the cells was observed as described above after staining with FDA. Cell viability was monitored by counting ∼800 FDA-positive cells with a Nageotte chamber 48 h after the heat treatment.

### Metabolite Extraction and Analysis by HPLC-ESI-MS

The metabolites were extracted, analyzed and annotated as previously described (Toffali et al., 2013). Briefly, packed cells collected by centrifugation were treated with four volumes of methanol containing 1% HCl (v/v) to extract the metabolites. After 20 min on ice, methanolic extracts were centrifuged twice to discard the cells and cell debris, and the samples were stored at -20°C. The methanolic extracts were diluted 1:4 with water (Fluka, Sigma–Aldrich), filtered with 0.2-μm pore mini-filters and injected (injection volume 10 μL) into a Beckman Coulter Gold 127 HPLC system (Beckman Coulter, Fullerton, CA, USA) equipped with an analytical Alltima RP C18 column (150 mm × 2.1 mm) and a C18 guard column (7.5 mm × 2.1 mm). The metabolites were eluted in a gradient of solvent A (90% (v/v) water, 5% (v/v) acetonitrile, 5% (v/v) formic acid), and solvent B (100% acetonitrile). The gradient started at 100% A and changed from 0–10% B in 5 min, 10–20% B in 20 min, 20–25% B in 5 min, 25–70% B in 15 min and back to 100% A in 1 min. The column was re-equilibrated for 20 min in 100% A. Each run was carried out twice at a flux rate of 0.2 mL/min.

Untargeted mass spectrometry (MS) was carried out using an ion-trap analyzer (Esquire 6000, Bruker Daltonics) equipped with an ESI source with nitrogen as the nebulizing gas (50 psi and 350°C) and the drying gas (10 L/min). MS was carried out in negative ion mode and the chromatogram data were acquired with Esquire v5.2 Control software (Bruker Daltonik GmbH, Bremen, Germany). The .d mass spectra data files were processed using Data Analysis v3.2 software (Bruker Daltonik GmbH). The analyzer was set up with the following parameters: target mass 400 m/z, ion scan 50–1500 m/z, and MS/MS and MS3 with 1 and 0.5 V of fragmentation amplitudes, respectively. Helium was used as the collision gas. To assess the reliability of metabolomics data, three biological replicates (i.e., three control or treated cell flasks or dishes) were always used in each experiment.

### Statistical Data Analysis

Exploratory data analysis was carried out by PCA whereas PLS regression was used to study the relationships between the proportion of cells containing cytoplasmic patches 24 h after heat treatment and variability in the abundance of specialized metabolites. In order to focus the analysis of the structured variation discovered by PLS on a small subset of significant metabolites, we applied VIP-based selection to PLS analysis (Chong and Jun, 2005). The threshold for the VIP parameter (Wold et al., 1993) to use in variable selection was estimated by maximizing the *Q*^2^ calculated for sevenfold full cross-validation (*Q*^2^_CV 7-fold_). To avoid overfitting and to prove the robustness of the models, we performed *N*-fold full cross-validation with different values of *N* (*N* = 6, 7, and 8) and the permutation test on the response (1000 random permutations) according to good practice for model validation.

Projection to latent structures VIP-based regression produced models with a small numbers of metabolites as predictors. To explore the role of these metabolites in more detail reducing the risk of overfitting and to improve the interpretation of the effects of heat stress in terms of metabolome variations, we extracted 100 random subsamples using the Monte Carlo method (probability of 0.70) and used the extracted subsamples as a training set for PLS VIP-based regression. The idea underlying this approach is that the relevant variables are always (or almost always) included in the models, whereas the others are selected in only a few cases corresponding to particular configurations produced by the sampling. As a consequence, the analysis of the spectrum of the most frequently used variables can provide a more detailed description of the metabolites related to heat stress, highlighting metabolites that act as markers for the level of damage produced by heat stress and integrating the findings generated by the initial phase of data analysis. Also, Monte Carlo sampling allowed the estimation of the error in prediction. Autoscaling was applied to the data set before analysis. PCA was carried out using SIMCA v13 (Umetrics, Umea, Sweden) whereas PLS VIP-based regression and Monte-Carlo subsampling were implemented as functions in the platform R v3.0.2 (R Foundation for Statistical Computing).

## Results

### Heat Stress Damage in Carrot Cells

**FIGURE 1:**
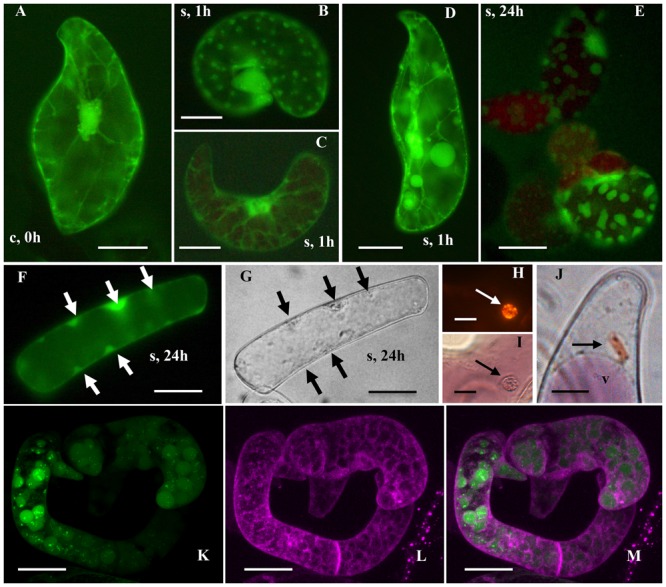
**Morphological features of control and heat-stressed cells.** Control **(A)** and heat stressed (44°C for 1 h) cells after 1 **(B–D)**, 24 **(E–J)**, and 96 **(K–M)** h of recovery. **(A–F)** Cells stained with 5 μg/mL FDA (green fluorescence); **(H)** and **(I)** cells stained with 1 μg/mL of Nile Red; **(K–M)** confocal images of a cell stained with 1 μM ER tracker blue–white (white fluorescence has been converted to magenta for clarity) and after 15 min with 5 μg/mL FDA. The arrows indicate the patches; v = vacuole. Bars: **(A–G)**,**(K–M)**: 50 μm; **(J)**: 30 μm; **(H)** and **(I)**: 20 μm.

Untreated R3M carrot cells showed the typical cytoplasmic organization, with a thin layer of peripheral cytoplasm, a large patch of cytoplasm containing the nucleus and many organelles, and a complex network of highly mobile cytoplasmic streams (**Figure 1A**). Heat treatment at 44°C for at least 30 min had a strong impact on the cytoplasmic organization, causing the disappearance of the cytoplasmic streams and their gradual replacement with static FDA-positive patches (**Figure 1B**; Supplementary Figure S1). When the cells were heat treated for 40 or 60 min, the modifications became more severe during subsequent cultivation at room temperature. Cells with small patches of cytoplasm instead of cytoplasmic streams became evident after 1 h at room temperature (**Figure 1B**), together with normal cells containing mobile streams (**Figure 1C**) as well as cells with streams and patches visible in the same cell (**Figure 1D**). Over the following hours and days, the number of cells with cytoplasmic patches rather than streams increased, and the patches became larger (**Figure 1E**). This morphology was also visible by bright-field microscopy (compare **Figures 1F,G**).

The nature of the patches was investigated by light and confocal microscopy and TEM. We found that they were cytoplasmic rather than vacuolar in origin due to the green rather than yellow FDA fluorescence (indicating a near neutral pH) and the absence of Lysotracker Red staining. Furthermore, the patches also enclosed various particles (**Figure 1K**), such as lipid droplets (as indicated by Nile Red staining in **Figures 1H,I**) and chromoplasts (**Figure 1J**). No mitochondria were observed, confirmed by the absence of Mitotracker^®^Green staining. Finally, the use of a probe specific for the ER (ER-tracker) showed that the cytoplasmic patches were surrounded by an ER membranes and thus may be nascent autophagosomes (**Figures 1K–M**).

**FIGURE 2:**
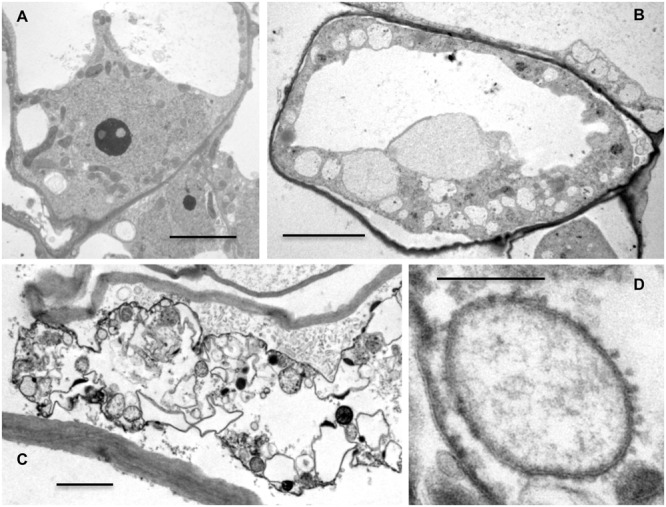
**Transmission electron microscopy of control and heat-stressed cells.** Pictures showing the ultrastructure of a control cell **(A)**, heat-stressed cell after 24 h **(B)** and 48 h **(C,D)** of recovery. Bars: 5 μm for **(A)** and **(B)**; 2 μm for **(C)**; 0.2 μm for **(D)**.

Transmission electron microscopy observations revealed that control cells had the characteristic cytoplasmic organization already observed in living cells, including a large main vacuole, a thin layer of peripheral cytoplasm, a large patch of cytoplasm containing the nucleus and many organelles (mainly mitochondria), and small additional vacuoles (**Figure 2A**). Heat-treated cells showed a variable morphology, including cells with a near normal appearance, cells showing evidence of minor plasmolysis (**Figure 2B**), and severely damaged cells with complete loss of plasma membrane and tonoplast integrity (**Figure 2C**). However, we did not observe complete cytoplasmic clearance (**Figure 2C**). The TEM images did not completely resolve the large FDA-positive structures surrounded by the ER membrane observed in the living cells, but we noted the presence of abundant small round structures surrounded by rough ER membranes (**Figure 2D**).

**FIGURE 3:**
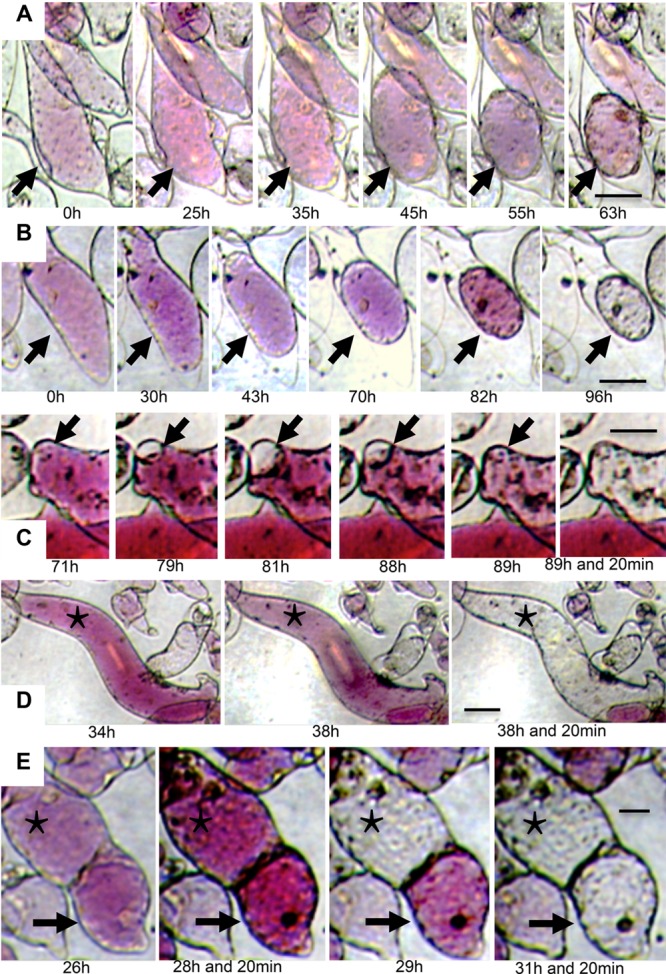
***In vivo*-imaging of pigmented heat-stressed R3M cells. (A)** and **(B)** show a slow cell death fate with irreversible plasmolysis; **(C)** shows reversible plasmolysis and sudden depigmentation; **(D)** and **(E)** show the typical cell death fate in this cell line. The arrows in **(A)** and **(B)** indicate cells committed to a slow cell death. The asterisks and arrows in **(C–E)** indicate cells that underwent rapid cell death. Bars: 50 μm for **(A,B,D)**, and **(E)**; 30 μm for **(C)**.

The fate of heat-stressed cells was investigated in living cultures by *in vivo*-imaging for 6 days. Following the application of heat stress for 1 h, a photogram was recorded every 20 min. Three replicate cultures were prepared allowing us to follow the fate of 194 cells, 176 of which died within the 6 days. The morphology of the heat-stressed cells was evaluated for the presence of cytoplasmic streams or patches 24 h after heat stress, and the characteristics of cell death were reported for non-surviving cells. Two cell death profiles were observed. The first involved slow plasmolysis over several days followed by rapid vacuolar depigmentation (**Figures 3A,B**), often preceded by transient deplasmolysis of the protoplasts (**Figure 3C**). The osmotic activity of the vacuole suggested the retention of tonoplast integrity (Ceoldo et al., 2005). The second profile involved the loss of mobility of the cytoplasmic streams accompanied by rapid vacuolar depigmentation (**Figures 3D,E**). This accelerated cell death has previously been described for cultured carrot cells and is the typical profile observed both during and at the end of normal culture cycles (Ceoldo et al., 2005). In neither case was cytoplasmic clearance observed.

The 18 cells that survived heat treatment during the 6 days of image recording were all normal cells with typical cytoplasmic streams, and no heat-induced cytoplasmic patches were evident. The effect of heat stress on these cells was limited to a transient slowing of the cytoplasmic streams followed by complete recovery before the end of the experiment (data not shown). None of the cells featuring heat-induced cytoplasmic patches survived the 6 days of observation and most of the cells containing these patches experienced the prolonged cell death profile. The imaging of living cells allowed the fate of each cell to be established unambiguously, revealing that cells with heat-induced cytoplasmic patches invariably experienced slow programmed cell death, whereas cells that retained their cytoplasmic streams could die slowly or rapidly, or could survive and recover completely.

**FIGURE 4:**
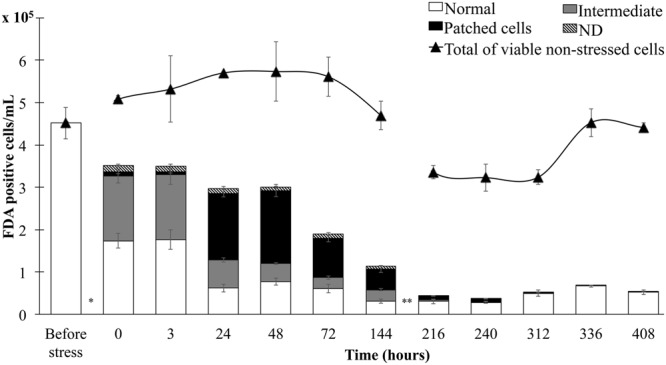
**Fluorescein di-acetate-positive control and heat stressed (1 h at 44°C) viable cells at different time points during recovery.** Unlike control cells, in which only the total sum is represented (triangle), the frequencies of the different morphologies of heat-stressed cells are shown. Column bars indicate the standard errors. ND, non-distinguishable. ^∗^Heat treatment 1 h at 44°C. ^∗∗^Growth medium refreshment (administration of 2 mL of fresh modified Gamborg B5 medium as described in the methods) after 150 h recovery.

The direct recording of living cells for several days has two main drawbacks. First, only a small number of cells can be followed (in our case 194 cells were followed in three experiments, corresponding to 18 days of total recording time). Second, the cells were immobilized in a thin layer of liquid medium under the microscope light for a long time which causes stress, resulting in a higher mortality rate (90.7%) than serial observations over the same period (74.7%). The morphology and fate of heated-stressed cells was therefore also followed by the serial observation of FDA-stained cell for 17 days (**Figure 4**). This revealed that cell death (i.e., the depletion of FDA-positive cells) occurred in three different periods: during the heat treatment (22%); between the 2nd and 3rd day after the heat treatment (24%) and between the 3rd and 6th days (17%); starting from the 2nd days, the cell death occurred concomitantly with an almost corresponding decrease of the cells with the patched cytoplasm, suggesting that the observed mortality is due to the death of the patched cells (**Figure 4**).

Toward the end of the experiments, we observed the complete recovery of surviving cells and the re-establishment of cell growth. These observations indicate that up to 22% of the cells do not respond favorably to heat stress (1 h at 44°C) and die during or immediately after the stress period. The remaining cells respond to heat stress in two ways: most undergo a gradual but complete rearrangement of the cytoplasm that ultimately leads to cell death, while a minority successfully recover and begin to proliferate as normal. In the latter case, visible heat damage was limited to the reversible slowing of cytoplasmic streams as observed by the *in vivo*-imaging experiments. Since abiotic stresses are known to cause ROS production in plant cells, attempts to measure ROS production and oxidation state in R3M cells were made, by loading cells with dichlorofluorescein diacetate (DCFH-DA) and by malondialdehyde (MDA) assay. Unpigmented cultures were used as control material, and the glucose/glucose oxidase H_2_O_2_ generating system was used as positive control during the setup of the method in the same cells. Unfortunately, ROS and lipid peroxidation measurement through MDA worked well only in unpigmented cells, while not repeatable results were obtained with R3M cell lines. This was probably due to the specific properties of ACs, that, being strongly colored and able to quench green fluorescence, interfere with the colorimetric and fluorimetric detection methods.

On the other side, when R3M cells were treated with the glucose/glucose oxidase H_2_O_2_ generating system, it caused cell death without the typical cytoplasm reorganization induced by heat treatment (not shown).

### Cytoskeletal Elements Are Functionally Damaged by Heat Stress

**FIGURE 5:**
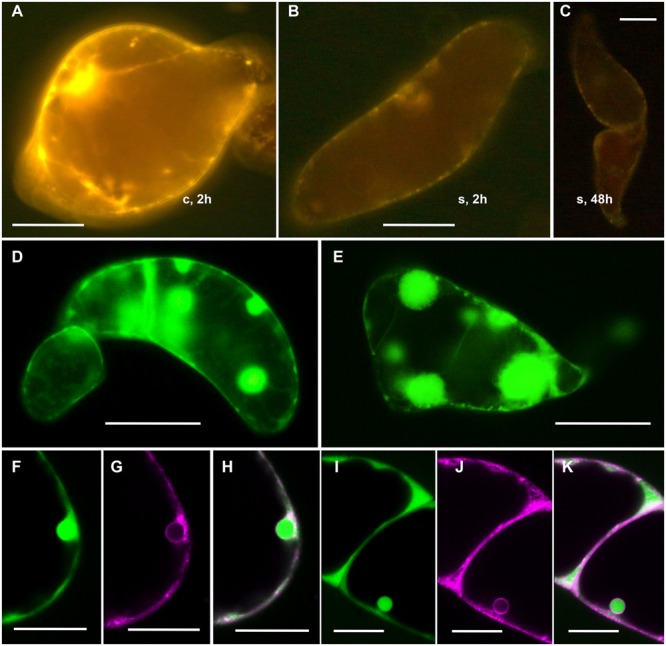
**Morphological features in heat-stressed and cytochalasin D treated cells.** Control **(A)** and heat stressed cells (after 2 h for **B** and 48 h for **C**) stained with 5 μg/mL FM1-43. **(D)** and **(E)** Cells treated with 0.05 mM cytochalasin D (24 h after treatment) stained with 5 μg/mL FDA. **(F–K)** Confocal microscopy of two different cells treated with 0.05 mM cytochalasin D (24 h after treatment) stained with both 1 μM ER tracker blue-white DPX (fluorescence in magenta; **G,J**) and, after 15 min, 5 μg/mL FDA (green fluorescence; **F,I**). **(H)** and **(K)** merge. Bars: **(A–E)** 50 μm; **(F–K)**: 20 μm.

The cytoskeleton is involved in the structure and mobility of plant cell cytoplasmic streams (Rogers and Gelfand, 2000; Shimmen and Yokota, 2004; Cai et al., 2015) so we used the endocytosis tracker FM-1-43 to investigate whether heat stress affects endocytosis and vesicle traffic, i.e., processes that require a functioning cytoskeleton. We found that FM-1-43 was distributed to most compartment membranes in untreated control cells within 2 h (**Figure 5A**) but the heat-stressed cells were unable to internalize or distribute the tracker either 2 h (**Figure 5B**) and 48 h (**Figure 5C**) after treatment. This suggests that the plasma membrane is not recycled by endocytosis after heat stress, consistent with the hypothesis of a damaged cytoskeleton. Even cells with normal morphology were unable to internalize the tracker after heat stress, indicating the existence of a gradient of heat-induced damage ranging from cells with apparently normal cytoplasmic organization but a loss of membrane flux through to cells showing visible changes in cytoplasmic organization.

To investigate the relationship between cytoskeletal damage and cytoplasmic reorganization, we exposed healthy unstressed cells to cytochalasin D and colchicine, which are known to damage microfilaments and microtubules, respectively. Some of the cells exposed to 0.05 mM cytochalasin D for 24 and 48 h exactly reproduced the morphological features of heat-stressed cells (**Figures 5D,E**), including cytoplasmic patches surrounded by an ER membrane (**Figures 5F–K**), but the only change observed following the treatment of cells with colchicine was a slight rounding of the cell shape. No remarkable morphological changes were observed at lower concentrations cytochalasin D, e.g., 0.01 mM as previously used by Lloyd and Traas (1988) and 0.02 mM as previously used by Traas et al. (1987). In contrast, the highest concentration we tested (0.2 mM) caused the rapid slowing of organelle movement and definitive arrest after 24 h (data not shown).

### The Modification of Specialized Metabolites Affects the Susceptibility of Cells to Heat Stress

The specialized metabolic profiles of cultured carrot cells can be modified in a targeted manner by feeding the cells with specific precursors (Toffali et al., 2013). The administration of coumaric, ferulic, or sinapic acids as acylation substrates resulted in the accumulation of the corresponding acylated ACs and some HCA derivatives, whereas feeding with caffeic acid had a broader impact on the specialized metabolome. No morphological changes were observed after HCA administration even after 48 h from the treatment. The ability of specific ACs and HCAs to protect carrot cells from heat stress was tested by feeding cells with different precursors prior to heat stress and comparing the morphological effects of heat stress to cells that were not provided with any precursors. The proportion of cells containing cytoplasmic patches 24 h after heat stress was chosen as a quantitative marker of susceptibility because these cells feature damaged cytoskeletal components and tend to undergo slow programmed cell death. We also treated cells with PIP as a further control, because this inhibits cinnamate 4-hydroxylase (Schalk et al., 1998) and thus blocks the hydroxylation of *trans*-cinnamic acid to *p*-coumaric acid, which is a precursor for the biosynthesis of ACs and other HCAs. This treatment prevented the accumulation of non-acylated ACs and ACs acylated with coumaric acid while promoting the accumulation of two new ACs acylated with cinnamic acid and PIP, respectively (Supplementary Figures S2 and S3, Supplementary Material 1).

**FIGURE 6:**
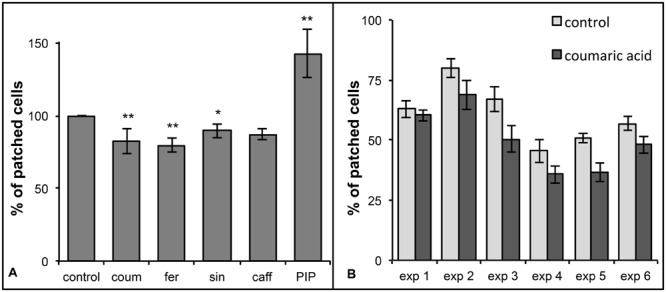
**Frequencies of cells containing cytoplasmic patches in heat-stressed cultures treated with HCA or PIP. (A)** Relative percentage of FDA-positive cells containing cytoplasmic patches in heat-stressed unfed (control) samples and cultures fed with 0.4 mM *p*-coumaric, ferulic, sinapic, or caffeic acids, and 0.1 mM PIP. The supplements were added to 12-day-old R3M cells, and after 24 h the fed cells were heat stressed at 44°C for 1 h. Cells were counted using a Nageotte chamber 24 h after the heat treatment. ^∗^*p* < 0.05, ^∗∗^*p* < 0.01. **(B)** Six independent experiments in which cells were fed with 0.4 mM *p*-coumaric acid and heat stressed after 24 h. Only FDA-positive cells containing cytoplasmic patches were counted using a Nageotte chamber after 24 h of recovery. With the exception of the first experiment, the frequencies of FDA-positive cells containing cytoplasmic patches in samples fed with 0.4 mM *p*-coumaric acid (dark gray columns) were lower than those assessed in unfed control samples (light gray columns).

The counting of cells containing cytoplasmic patches is a time consuming process, so we carried out many independent experiments to ensure sufficient data were available for analysis. In each of the 16 independent experiments with HCAs as precursors, the treated samples featured a lower proportion of damaged cells than the control samples (**Figure 6A**). In five further experiments with PIP, the treated cells showed a higher proportion of damaged cells than the control samples (**Figure 6A**).

Carrot cells in liquid medium have a dynamic specialized metabolome, so that visual selection of cells accumulating ACs is necessary to keep the pigmented phenotypical trait (Ceoldo et al., 2009). Probably as a consequence, also the susceptibility of untreated cells to heat stress changed over time; thus, data normalization was necessary to compare the different experiments. **Figure 6A** shows the proportion of damaged cells in the treated samples relative to the controls, whereas **Figure 6B** shows the variability between experiments in which the cells were fed with *p*-coumaric acid, as a representative example.

### Only Certain Metabolites Confer Protection against Heat Stress

To investigate which specific individual specialized metabolites confer protection against heat stress, carrot cell cultures fed with HCA precursors were compared to controls using an untargeted metabolomics strategy based on HPLC-ESI-MS. The cells were analyzed after feeding but just before heat stress application to determine whether specific metabolites confer protection from heat stress, and 24 h after heat stress to determine whether cells can respond to stress by modifying the specialized metabolome.

**FIGURE 7:**
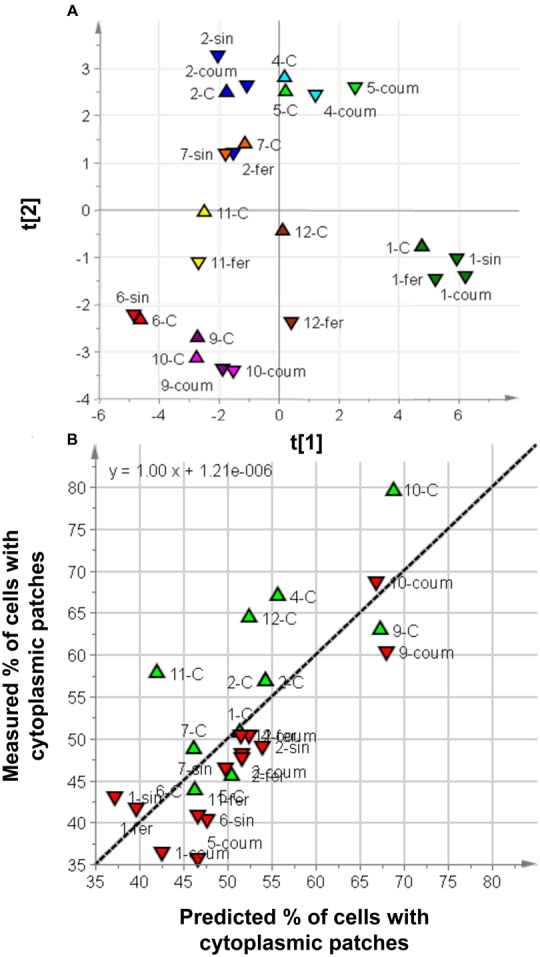
**Multivariate analysis of relationships between metabolites and heat-induced cell damage. (A)** Score scatter plot for the PCA model of the collected samples described according to the specialized metabolome (triangles = controls, inverted triangles = treated cells). Different colors are used to indicate the experimental sessions. **(B)** VIP-based PLS regression model for the collected samples: predicted vs. measured proportion of cells containing cytoplasmic patches.

The metabolic profile of the cultures after feeding and just before the application of heat stress confirmed our previous findings (Toffali et al., 2013) and these data are summarized in Supplementary Figure S4. Feeding with *p*-coumaric, ferulic, and sinapic acids caused targeted metabolomic changes, i.e., the corresponding ACs became more abundant as well as other metabolites derived from the same HCAs. Feeding with caffeic acid also induced multiple untargeted effects on the specialized metabolome and the caffeic acid feeding experiments were therefore excluded from subsequent analysis. A data matrix was built combining the proportion of damaged cells in each experiment with the relative levels of individual metabolites and groups of metabolites, as determined by HPLC-ESI-MS. PCA explained 63% of the variance in metabolite levels but did not highlight the presence of outliers on the basis of the Hotelling’s T2 test and the DModX test at the 99% level. The dominant effect of the experimental session on the metabolic variance is shown by the clustering in the score scatter plot (**Figure 7A**). Feeding with *p*-coumaric, ferulic, and sinapic acids caused a significant reduction in the proportion of cells with cytoplasmic patches compared to controls (*p* < 0.001).

The observation that all the feeding treatments were effective in preventing heat induced cell damage suggests that various metabolites are active in cell protection from heat stress.

By limiting our analysis to the unfed control cells, a reliable regression model was built by PLS VIP-based regression using only three metabolites. One coumaric acid hexose (coumaric acid h.2), the ferulic acid hexose (ferulic acid h.) and the caffeic acid acylated cyanidins (Cy(caf)tot) yielded a PLS model with one component (*R*^2^ = 0.60, *Q*^2^_CV 7-fold_ = 0.36, error in calculation = 6.2, estimated error during sevenfold full cross-validation = 8.0, and error in prediction calculated by Monte Carlo sampling = 8.5). Monte-Carlo sampling was unable to add relevant metabolites to the PLS VIP-based analysis.

On the other hand, when only the fed cells were considered in the data modeling, a reliable PLS VIP-based regression model (*R*^2^ = 0.65, *Q*^2^_CV 7-fold_ = 0.43, error in calculation = 5.1, estimated error during sevenfold full cross-validation = 6.5, and error in prediction by Monte Carlo sampling = 6.3) was obtained using coumaric aspartic acid (coumaric aspartic acid-1), coumaric acid hexose (coumaric acid h.2) and caffeic acid acylated cyanidins (Cy(caf)tot.) Monte Carlo sampling did not add other relevant variables.

By considering the entire dataset (fed and unfed cells), a reliable regression model was built by PLS VIP-based also using only three metabolites. One coumaric aspartic acid (coumaric aspartic acid-2), the caffeic acid acylated cyanidins (Cy(caf)tot.) and the dicaffeic daucic acid yielded a PLS model with two components (*R*^2^ = 0.60, *Q*^2^_CV 7-fold_ = 0.49, error in calculation = 6.7, estimated error during sevenfold full cross-validation = 7.1, and error in prediction by Monte Carlo sampling = 7.4). **Figure 7B** shows the plot of predicted vs. measured proportions of cells with cytoplasmic patches. Monte-Carlo sampling highlighted a subset of five metabolites or groups related to heat stress protection. Specifically, one coumaric aspartic acid (coumaric aspartic acid-1), the combination of all coumaric acid derivatives (tot. Coum.a.d) and the caffeic acid acylated cyanidins (Cy(caf)tot.) showed a strong inverse relationship with the proportion of damaged cells, whereas dicaffeic daucic acid and the other coumaric aspartic acid (coumaric aspartic acid-2) were only weekly related to the proportion of damaged cells.

The metabolites and groups common to the three regression models included the two caffeic acid acylated cyanidins (Cy(caf)tot.) as well as several coumaric and caffeic acid derivatives, which are therefore likely to be closely involved in the protection of cells against heat stress.

The metabolomic profile of the cells after heat stress should reveal the natural ability of cells to respond to heat stress by modifying specific metabolites. The total signal (sum of the signal intensities representing all of the metabolites we detected) offers a rough indication of the total metabolite content, and this declined by an average of ∼20% after heat stress, probably reflecting the cell death we observed (see above). In addition to the general loss of metabolites, heat stress specifically induced the accumulation of three compounds: free caffeic acid and two distinct coumaric quinic acids. These molecules were barely detectable in the unstressed samples, but their abundance increased by at least twofold and in some cases by up to 16-fold in the samples after heat stress (Supplementary Figure S5).

## Discussion

The precise biological role of specific specialized metabolites can be challenging to define because it is difficult to establish biological systems (such as whole plants and even cell suspension cultures) that differ solely in the content of individual molecules. This reflects the ability of plant cells to accumulate diverse metabolites by the decoration of a smaller number of basic structures, but the precise meaning of the existence of this plethora of different decorations *in vivo* as well as how they are genetically regulated and obtained is poorly understood. Although the specialized metabolome can be modulated by the overexpression of structural and regulatory genes in many species, this usually does not achieve the precision required to investigate individual metabolites. As an example, carrot cells accumulate non-acylated ACs and ACs acylated with coumaric, caffeic, ferulic, and sinapic acids. The biosynthesis of the acylated ACs in carrot cells is catalyzed by acyltransferases, which are remarkably promiscuous in terms of their organic acid substrates, to the extent that exogenously supplied 3,4,5 trimethoxycinnamic acid can be incorporated into ACs even though this is not produced naturally in carrot cells (Baker et al., 1994; Dougall et al., 1998). The accumulation of individual ACs therefore strongly depends on the availability of acylation substrates, which is difficult to control by genetic manipulation.

One way to address the substrate-dependent acylation of ACs is by feeding the cells with specific HCAs. These are taken up by the cells to generate an abundant substrate reservoir which favors the formation of the corresponding acylation products as well as other related HCA derivatives (Toffali et al., 2013). On the other hand, the administration of HCAs could determine a shift from a more heat shock permissive to a heat shock tolerant stage of the cell cultures. Indeed, feeding could trigger ROS production, thus inducing the cells to be more tolerant against various abiotic stresses. Although the levels of the administered HCAs were quite low and the short time from feeding and heat treatment (24 h) did not determine visible morphological effects, they could have stressed the cells as well. However, the evidence that the different treatments gave heat protection with different efficiency suggests that feeding conferred heat tolerance due to the different precursor internalization, conversion and finally accumulation of few specific specialized metabolites rather than the putative (not observed in our experiments) metabolic wide modification that could occur during the stress tolerance process (described for instance by Kaplan et al., 2004).

We decided to use this approach to change the specialized metabolome in carrot cells in a targeted manner, allowing us to investigate the specific roles of individual ACs and HCA derivatives in the context of abiotic stress.

### Heat Stress Triggers Programmed Cell Death in Carrot Cells, Marked by Specific Cell Morphology

We established a stress response screening platform based on carrot cell cultures accumulating ACs and sought quantifiable markers of cell damage. Various forms of stress were tested, including mechanical stress, osmotic stress, starvation and extreme temperatures. Heat treatment at 44°C for 1 h caused short-term and long-term damage in these cells. Approximately 22% of the cells were killed almost immediately by the heat treatment, and most of the remaining cells died during the subsequent 6 days. Only ∼10% of the cells survived and recovered completely. Therefore, although the heat treatment was severe enough to cause massive cell death, a significant proportion of cells recovered from the damage. Most of the cells died slowly due to the accumulation of damage over several days, which was made apparent by the gradual replacement of the typical mobile cytoplasmic streams with static cytoplasmic patches that increased in size over time. In cells containing such patches, death was usually preceded by gradual protoplast shrinkage lasting several days, which is not the case when cells die under normal cultivation conditions (Ceoldo et al., 2005).

Cytoplasmic clearance was not detected by TEM, so the slow cell death profile we observed was dissimilar to previously reported non-autolytic and non-vacuolar processes yet the delay before the loss of plasma membrane integrity clear ruled out necrosis (Van Doorn, 2011; Van Doorn et al., 2011). Like other non-autolytic processes such as victorin-induced cell death (Curtis and Wolpert, 2004), the process we observed was characterized by cytoplasmic shrinkage without permeabilization of the plasma membrane (as demonstrated by the fluorescence of the cytoplasmic patches after FDA staining) and without tonoplast rupture (as demonstrated by the osmotic activity of some cells just before the final collapse, and by the absence of AC-specific red autofluorescence in the cytoplasm of pigmented cells after heat stress). Cell death characterized by protoplast shrinkage was previously reported in cultured carrot cells as the result of mild heat stress, whereas intense heat stress caused cell death without any shrinkage (McCabe et al., 1997; Reape et al., 2008).

Actin microfilaments were probably damaged soon after the onset of stress, as suggested by the immediate and almost complete inhibition of endocellular movement/endocytosis and by the disappearance of cytoplasmic streaming, both of which depend on actin microfilaments (Kim et al., 2005; Verchot-Lubicz and Goldstein, 2010). The administration of cytochalasin D, which promotes F-actin depolymerization (Kim et al., 2012), mimicked the damage caused by heat stress, supporting the role of microfilament damage in heat-induced cell death. Actin microfilaments have several roles during programmed cell death in plants. A functional polymerized actin microfilament system is required for programmed cell death in *Picea abies* somatic embryo suspension cultures (Smertenko et al., 2003) whereas actin depolymerization induced by calcium ions initiates programmed cell death in *Papaver* spp. pollen during the self-incompatibility response (Bosch and Franklin-Tong, 2008). The FDA-positive cytoplasmic patches in the heat-stressed carrot cells were surrounded by ER membranes and contained particles such as lipid droplets and chromoplasts, partially resembling the structure of autophagosomes (Li and Vierstra, 2012). Alternatively, these structures could reflect the loss of structural organization in the cytoplasm following microfilament damage. In either case, our live cell imaging and serial analysis of heat-stressed cells indicated these structures were useful markers of cell death, especially the programmed cell death profile involving slow plasmolysis.

### Metabolites Containing Coumaric and Caffeic Acid Correlate with Heat Stress Protection

We investigated the correlation between heat-induced damage, represented by the proportion of cells with cytoplasmic patches, and individual metabolites that accumulated just before the stress was applied, as determined by HPLC-ESI-MS. Correlations were defined by VIP-based PLS regression analysis using a panel of 16 independent experiments in which the cells were fed with different HCAs. We also investigated the relationships between the levels of different metabolites or groups of metabolites and heat stress protection in control cells. Our rationale for this approach was that feeding induces both resistance to heat stress and the accumulation of a small groups of metabolites, thus we would not be able to discriminate between more or less active metabolites in fed cells because all the metabolites would correlate with cell protection. Furthermore, the metabolome of cultured cells changes over time which could affect the ability of control cells to respond to heat stress in different experiments. We therefore looked for evidence of a relationship between heat-induced cell damage and metabolites or group of metabolites also in the unfed control cells.

The analysis of control samples and all samples (fed and unfed cells) revealed relationships between the levels of specific metabolites and the prevention of heat stress damage both in the treated and control samples. In the controls, the differing abundance of the metabolites reflects the low stability of the productive traits in this cell line, resulting in a dynamic metabolome. In the fed cells, the differing abundance of the metabolites reflects the targeted feeding strategy and hence the presence of particular precursors. Our analysis also showed that the modification of the metabolome by feeding can reduce damage caused by heat stress; the analysis of the whole dataset and the control dataset highlighted only two caffeic acid acylated ACs (Cy(caf)tot) and a small number of coumaric and caffeic acid derivatives as metabolites with potential key roles in heat stress protection.

Untargeted metabolomics analysis showed that the levels of most metabolites declined sharply after heat stress, probably due to cell death and subsequent metabolite degradation, but the abundance of three metabolites that were barely detectable in unstressed cells (free caffeic acid and two coumaric quinic acids) specifically increased in response to heat stress. This does not prove their active involvement in protection, but because molecules containing caffeic and coumaric acids (including ACs) showed a strong correlation in the prevention of heat stress, the fact that cells accumulated these molecules in response to heat stress further strengthens this hypothesis. The heat-damaged cells showed evidence of profound structural disorganization which is unlikely to be compatible with biosynthesis activity, so the accumulation of these protective molecules could be restricted to the few cells that survive. However, the possibility that free caffeic acid accumulates due the degradation of other caffeic acid-containing metabolites cannot be excluded.

Heat stress is known to induce the production of phenolic compounds in plants (Wahid et al., 2007) but few reports have considered the role of individual metabolites. The accumulation of total phenols under heat stress has been reported in tomato and watermelon plants, where it correlates with an increase in phenylalanine ammonia lyase activity and a decrease in peroxidase and polyphenol oxidase activities, probably as part of the acclimation to heat (Rivero et al., 2001; Bita and Gerats, 2013). ACs can accumulate or decline in response to high temperatures (Wahid et al., 2007). They tend to accumulate in vegetative tissues (Wahid and Ghazanfar, 2006) but decline under the same conditions in reproductive organs (Tomana and Yamada, 1988; Shaked-Sachray et al., 2002). ACs may reduce the osmotic potential of leaves under high temperature conditions, which in turn increases water uptake and reduces transpiration loss (Chalker-Scott, 2002). In contrast, the application of foliar caffeic acid to heat-stressed *Gossypium arboreum* plantlets, which are particularly heat sensitive, reduced electrolyte leakage from the cotyledon leaf and promoted the activities of α-amylase and β-amylase, which are involved in heat stress protection (Thind and Barn, 2012). Martinez et al. (2016), in a detailed metabolomics analysis of heat stressed tomato, showed heat induced accumulation of free coumaric acid and *p-*coumaroyl-CoA and various flavonols, and heat-induced depletion of other HCAs as dicaffeoyl quinic, caffeoyl feruloyl quinic acids. We have demonstrated a role of phenylpropanoids in the prevention of heat-induced cell death, and we provide evidence that certain metabolites (caffeoylated cyanidins and few coumaric and caffeic acid derivatives) might play a specific protective role.

### Potential Protective Mechanisms of Key Metabolites

Heat stress has different effects on cells depending on the nature of the stress. Extreme high temperatures cause almost instantaneous cell death whereas moderately high temperatures cause protein denaturation and aggregation, and affect RNA stability, membrane fluidity and integrity (Wahid et al., 2007). We found that microfilament integrity was affected by heat stress, as previously reported by Müller et al. (2007) who showed that *Arabidopsis thaliana* epidermal root cells experienced a transient disruption of microtubule and microfilament structures within 1–3 h after a 40–41°C heat shock followed by recovery at 20°C. Malerba et al. (2010) observed the same cytological events in tobacco BY-2 cells exposed to 50°C for 5 min.

Heat stress, like other forms of abiotic stress, also stimulates the production of ROS in plant cells (Bita and Gerats, 2013). Phenylpropanoid compounds such as ACs and HCAs are well-known ROS scavengers (Ferreres et al., 2011; Nakabayashi et al., 2014). Unfortunately, in this work the attempts to directly measure ROS and/or the oxidative status of the fed and unfed heat-treated cells were unsuccessful, thus preventing to give a clear answer on the ROS scavenging role of the metabolites accumulated after the feeding with the molecular precursors. However, the observation that oxidative status induced by the glucose/glucose oxidase H_2_O_2_ generating system caused cell death without the typical cytoplasm reorganization induced by heat treatment, suggests that these typical modifications, that in these cells anticipated the cell death induced by heating, could not simply be a direct effect of the ROS increase. ROS can directly damage a wide range of cellular components, including mitochondria (Taylor and Millar, 2007), chloroplasts (Khanna-Chopra, 2012), and cytoskeletal components such as microtubules (Livanos et al., 2014). We found that heat stress caused the disruption of microfilaments, so the putative protection of this cytoskeletal component by specific phenylpropanoids and flavonoids from heat stress could reflect a mode of ROS detoxification that has yet to be characterized, or could reflect independent and ROS unrelated microfilament protection mechanisms. Microfilament damage by ROS has yet to be reported in plants, although this mechanism is well known in yeast and is linked to the induction of apoptosis (Perrone et al., 2008).

Certain flavonoids can influence the polymerization of microfilaments *in vitro* (Böhl et al., 2007), e.g., kaempferol and fisetin reduce the polymerization rate of animal actin in a dose-dependent manner, whereas quercetin has the weakest inhibitory effect and epicatechin even promotes polymerization. Genistein and the cyanidin precursor taxifolin inhibit actin polymerization at relatively high concentrations (25 μM) but have no effect at lower concentrations (Böhl et al., 2007). These findings suggest that these phenolic compounds have additional roles beyond their antioxidant activity.

The vacuole, where many specialized metabolites accumulate, is physically separated from ROS generation centers, such as chloroplasts and peroxisomes. However, some evidences showed that the hydroperoxyl radical and hydrogen peroxide (H_2_O_2_) could freely cross the membrane (Yamasaki et al., 1997) and that tonoplast aquaporines might regulate the flux of H_2_O_2_ through the tonoplast membrane, allowing it to reach the vacuolar sap (Bienert et al., 2007; Maurel et al., 2009). Since R3M cells have central vacuoles that occupy the most part of the entire volume of the cells, it could be speculated the produced ROS could cross the tonoplast through aquaporines or others, not yet characterized, systems and being detoxified by vacuolar ACs and HCA derivatives. The presence of the vacuolar peroxidase (POX)/dihydroxyflavonoids/ascorbate (ASC) system, responsible for the conversion of oxidated flavonoids into the original form (Yamasaki et al., 1997), and the knowledge that the detoxification rate of H_2_O_2_ mediated by this system is very closed to the H_2_O_2_ generation rate (Agati et al., 2013), suggest that vacuole might be an active compartment, where ROS might be detoxified.

Most investigations describing quantitative correlations between classes of metabolites and stress have not focused on individual compounds, but have instead described global metabolic changes. For example, 143 different metabolites were modulated in *A. thaliana* plants subjected to heat shock at 40°C for up to 240 min, but individual compounds were not considered (Kaplan et al., 2004). The only studies that have measured individual metabolites involve responses to UV-B stress. Li et al. (1993) showed that *A. thaliana* chalcone synthase (*CHS*) mutants do not accumulate kaempferol derivatives and are highly sensitive to UV-B exposure, whereas *A. thaliana* chalcone isomerase (*CHI*) mutants do not synthetize kaempferol derivatives or significant levels of sinapate esters and are even more UV-B sensitive than the *CHS* mutants, demonstrating that kaempferol and sinapic acid derivatives both play a role in UV-B protection. In agreement with these data, Bieza and Lois (2001) isolated a mutant that tolerated lethal doses of UV-B from a population of UV-B-sensitive mutants subjected to chemical mutagenesis, and found that UV-B tolerance was accompanied by an increase in the abundance of flavonoids and one sinapate derivative known to absorb UV-B radiation.

## Author Contributions

FG designed the research and wrote the paper. ML contributed to the experiment design. MC and KT performed most of the experimental work. MC assisted with paper writing and provided the figures and tables. SC gave technical assistance in the feeding experiments. PS performed the PIP experiments. MS supervised the statistical analysis. BB performed the TEM experiments.

## Conflict of Interest Statement

The authors declare that the research was conducted in the absence of any commercial or financial relationships that could be construed as a potential conflict of interest.

## Footnotes

1 http://imagej.nih.gov/ij

## Abbreviations

AC: anthocyanin
DMSO: dimethylsulfoxide
ER: endoplasmic reticulum
FDA: fluorescein di-acetate
HBA: hydroxybenzoic acid
HCA: hydroxycinnamic acid
HPLC-ESI-MS: high performance liquid chromatography-electrospray ionization-mass spectrometry
PCA: principal component analysis
PIP: piperonylic acid
PLS: projection to latent structures
ROS: reactive oxygen species
TEM: transmission electron microscopy
