# Supplementary material for: Impact of Phenylpropanoid Compounds on Heat Stress Tolerance in Carrot Cell Cultures
Source: Front Plant Sci. 2016 Sep 22;7:1439. doi: 10.3389/fpls.2016.01439 (PMC5031593; doi:10.3389/fpls.2016.01439)
Supplement: Supplementary file 1 [file Data_Sheet_1.PDF]

Supplementary Material

Impact Of Phenylpropanoid Compounds On Heat Stress Tolerance In Carrot Cell Cultures

Mauro Commisso[$], Ketti Toffali[$], Pamela Strazzer, Matteo Stocchero, Stefania Ceoldo, Barbara Baldan, Marisa Levi and Flavia Guzzo[*]

[$]These authors equally contributed to the manuscript

[*]**Correspondence:**
Corresponding Author: Flavia Guzzo
Email Address: flavia.guzzo@univr.it

Supplemental Figure 1

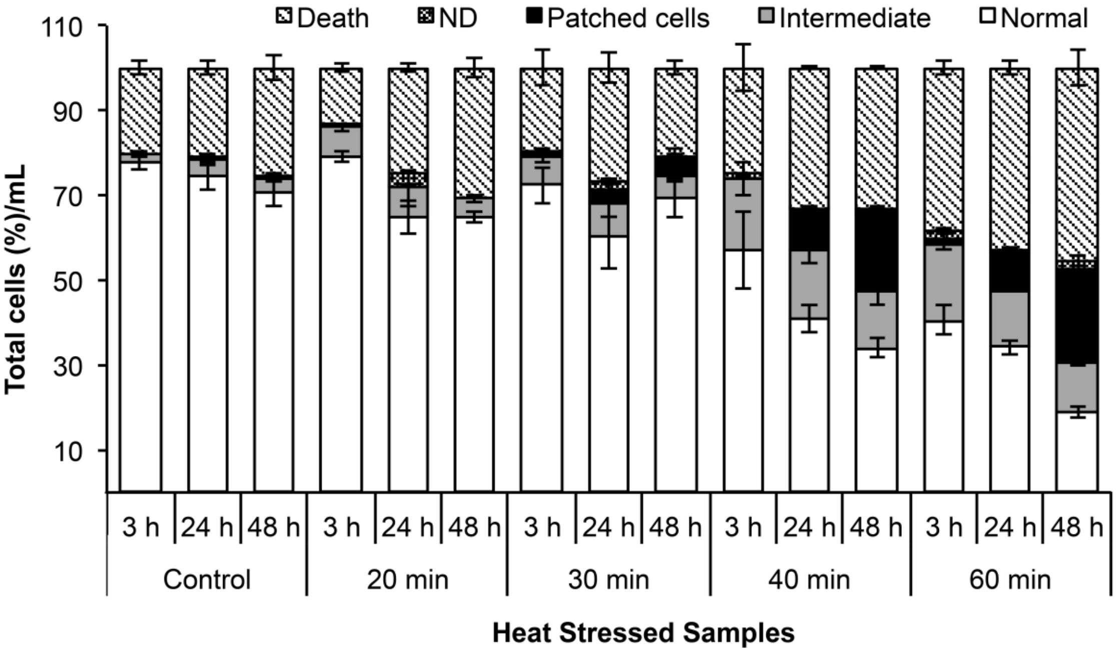

Figure S1. Frequencies of the different cell morphologies in samples heat stressed at 44°C for different times. The number and morphology of cells were determined using a Nageotte counting chamber. Each measurement was carried out three times. Viable cells and their morphologies were determined after staining with 5 µg/ml FDA. ND, non-distinguishable. Error bars: standard errors.

Supplemental Material 1

Further results from PIP treatment.
Treatment with PIP modified the R3M metabolome without affecting cell viability (data not shown). Compared to the typical AC ratios, PIP treatment reduced the levels of specific ACs (Figure S2A, peaks 2 and 5). HPLC-ESI-MS analysis showed that PIP reduced the accumulation of non-acylated ACs, and mainly cyanidin pentose hexose and cyanidin (coumaric acid) pentose hexose hexose among

the acylated ACs (Figure S2B). This result was expected because PIP is an inhibitor of cinnamate 4-hydroxylase (C4H) and therefore blocks the hydroxylation of *trans*-cinnamic acid to *p*-coumaric acid, which is in turn a precursor for the biosynthesis of ACs and other phenylpropanoids such as the HCAs also used for AC acylation.

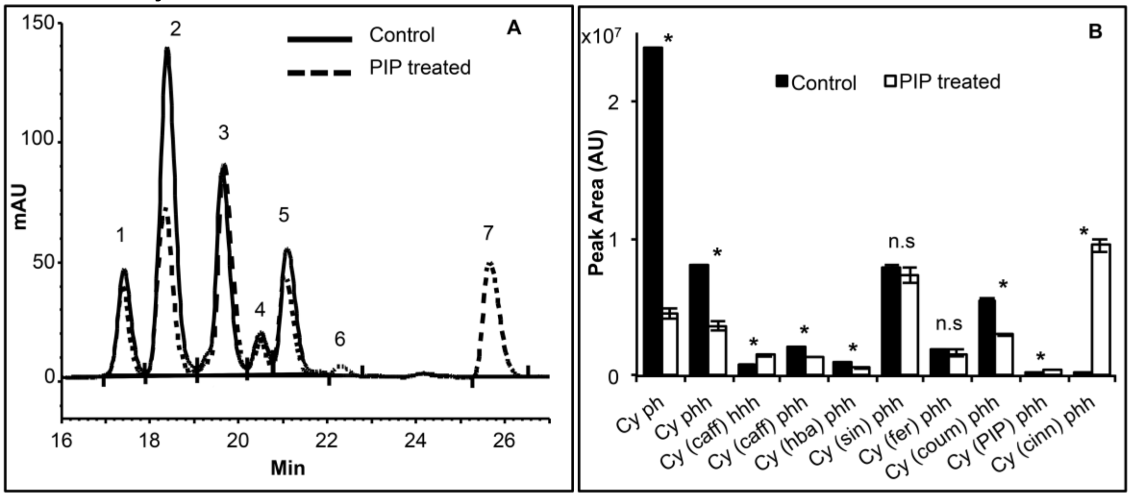

Figure S2. The effect of PIP treatment on the AC profile of R3M cells. A) Two HPLC-DAD chromatograms at 520 nm showing representative samples of the R3M cell line after treatment with PIP (dashed line) compared to untreated control cells (solid line). Peak numbers are annotated as follows: (1) Cy p.h.h., (2) Cy p.h. + Cy (caff) h.h.h. + Cy (caff) p.h.h., (3) Cy (hba) p.h. + Cy (sin) p.h.h., (4) Cy (fer) p.h.h., (5) Cy (coum) p.h.h., (6) Cy (PIP) p.h.h., (7) Cy (cinn) p.h.h. Abbreviations: Cy, cyanidin; caff, caffeic acid; hba, hydroxybenzoic acid; sin, sinapic acid; fer, ferulic acid; coum, coumaric acid; p and h for pentose and hexose, respectively. B) Abundance of different ACs, expressed as the signal intensity in arbitrary units (AU), evaluated by HPLC-ESI-MS in cells treated with PIP and untreated controls. Error bars: standard errors; *$p < 0.01$; n.s = not significant.

PIP treatment also affected the accumulation of two different ACs (Figure S2A, peaks 6 and 7) that were identified by HPLC-ESI-MS as cyanidin (PIP) pentose hexose hexose and cyanidin (cinnamic acid) pentose hexose hexose. These molecules do not usually accumulate in R3M cells (Figure S2B), showing that the cells can use unnatural substrates for the biosynthesis of corresponding acylated ACs due to the known promiscuity of carrot acyltransferases (Baker et al. 1994). However, the potential biological activity of these novel ACs is difficult to predict due to the biochemical properties of the unnatural acyl groups. For example, the well-known ability of HCAs to scavenge ROS depends on the number of hydroxyl groups on the aromatic ring (Kikuzaki et al. 2002). The novel ACs in the carrot cells were acylated with PIP and cinnamic acid, neither of which possess hydroxyl groups on the aromatic ring, so they could have unanticipated biological activities or even no activity at all.

PIP also caused a reduction in the levels of specific non-AC-linked coumaric acid derivatives, the accumulation of the glycosylated and aspartated forms of cinnamic acid, and (surprisingly) the accumulation of PIP (Figure S3A,B). These findings confirm that PIP is an effective inhibitor of C4H and also that carrot cells may exploit certain xenobiotic substances as enzymatic substrates (Baker et al. 1994). Furthermore, the amounts of the most abundant molecules in the R3M metabolome, i.e. dicaffeoyl daucic acid, caffeoyl quinic acid, dicaffeoyl quinic acid and caffeoyl quinic acid methyl derivatives, were strongly reduced by the treatment with PIP, probably reflecting the low abundance of the coumaric acid derivative precursors (Figure S3C).

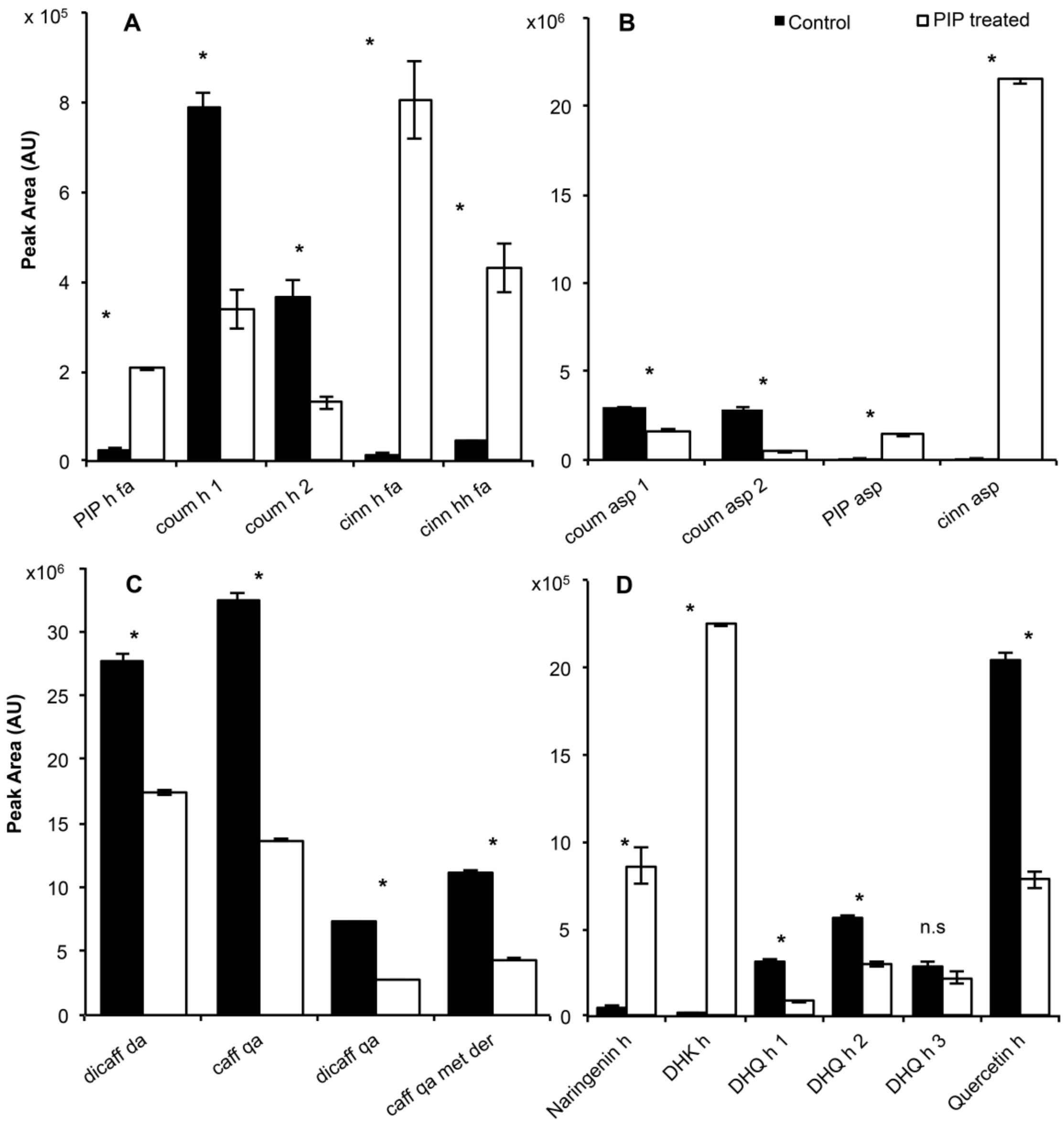

Figure S3. The effect of PIP treatment on other R3M metabolites. The metabolite levels expressed as arbitrary units (AU) were determined by the HPLC-ESI-MS analysis of treated and untreated cells. Error bars: standard errors; * $p < 0.01$, n.s = not significant. Abbreviations analysis of treated and untreated cells. Error bars: standard errors; * $p < 0.01$, n.s = not significant. Abbreviations: coum, coumaric acid; cinn, cinnamic acid; caff, caffeic acid; h, hexose; fa, formic acid adduct; dicaff da, dicaffeoyl daucic acid; caff qa, caffeoyl quinic acid; dicaff qa, dicaffeoyl quinic acid; caff qa met der; caffeoyl quinic acid methyl derivative; p, pentose; sin, sinapic acid; asp, aspartic acid; DHK, dihydrokaempferol; DHQ, dihydroquercetin.

Flavonoids such as dihydroquercetin and quercetin derivatives also accumulated at lower than normal levels in R3M cells after treatment with PIP, whereas naringenin and dihydrokaempferol derivatives (which represent earlier steps in the pathway) accumulated to higher levels than usual (Figure S3D). These compounds belong to the cellular flavonoid pool and their transformation requires specific hydroxylation steps. The synthesis of dihydrokaempferol from naringenin requires the hydroxylation of carbon-3 in the C-ring, catalyzed by flavanone 3-hydroxylase (F3H), a 2-oxoglutarate-dependent

dioxygenase isolated and characterized for the first time from *Petroselinum crispum* (Britsch, 1990). The synthesis of dihydroquercetin from dihydrokaempferol requires the hydroxylation of carbon-3' in the B ring, catalyzed by flavonoid 3'-hydroxylase (F3'H), a cytochrome P450 enzyme. The corresponding step in carrot cells appears to be partially blocked, resulting in the accumulation of the upstream pathway intermediates naringenin and dihydrokaempferol, at the expense of the downstream products dihydroquercetin and quercetin.

Supplemental Figure 4

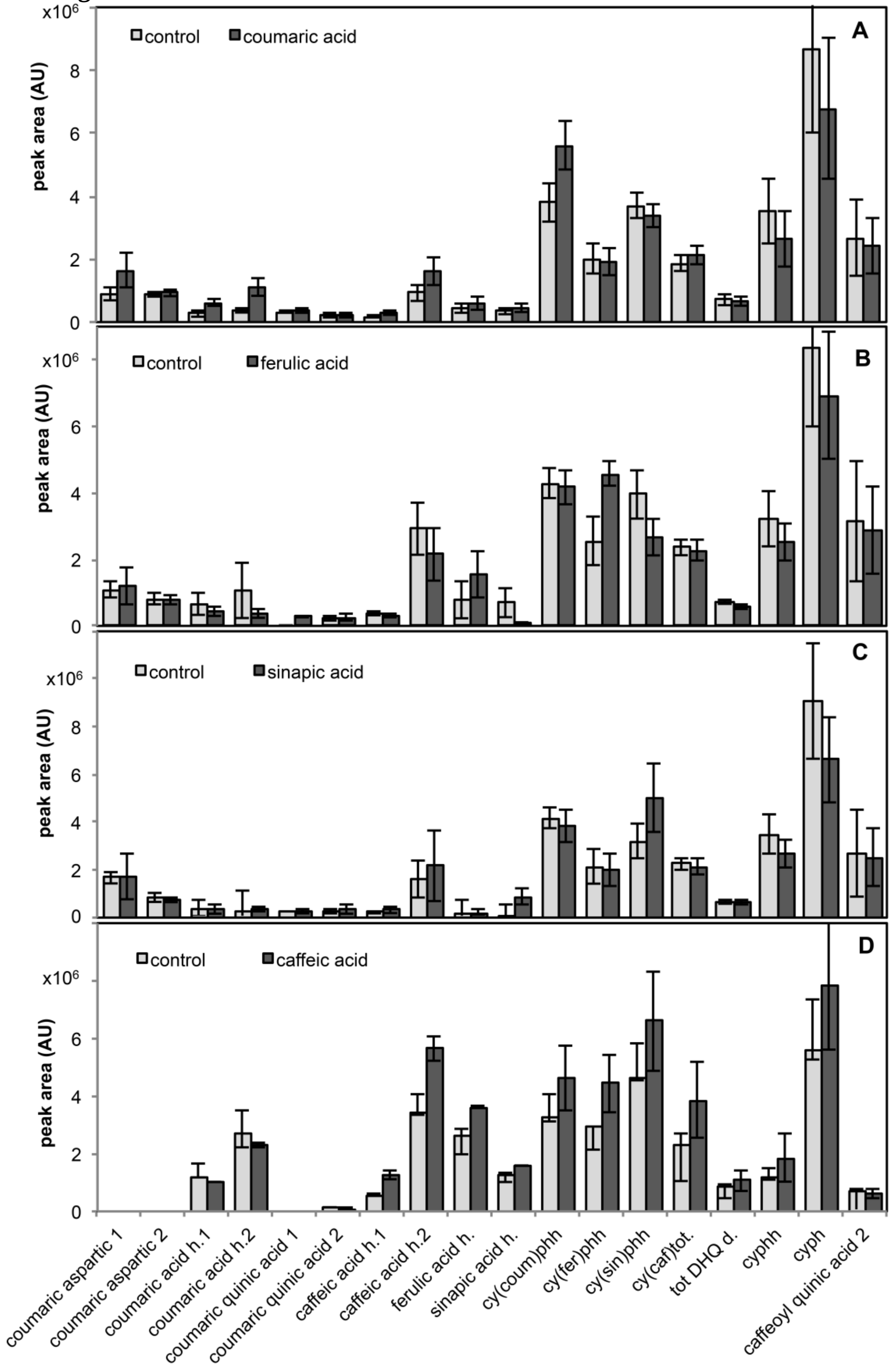

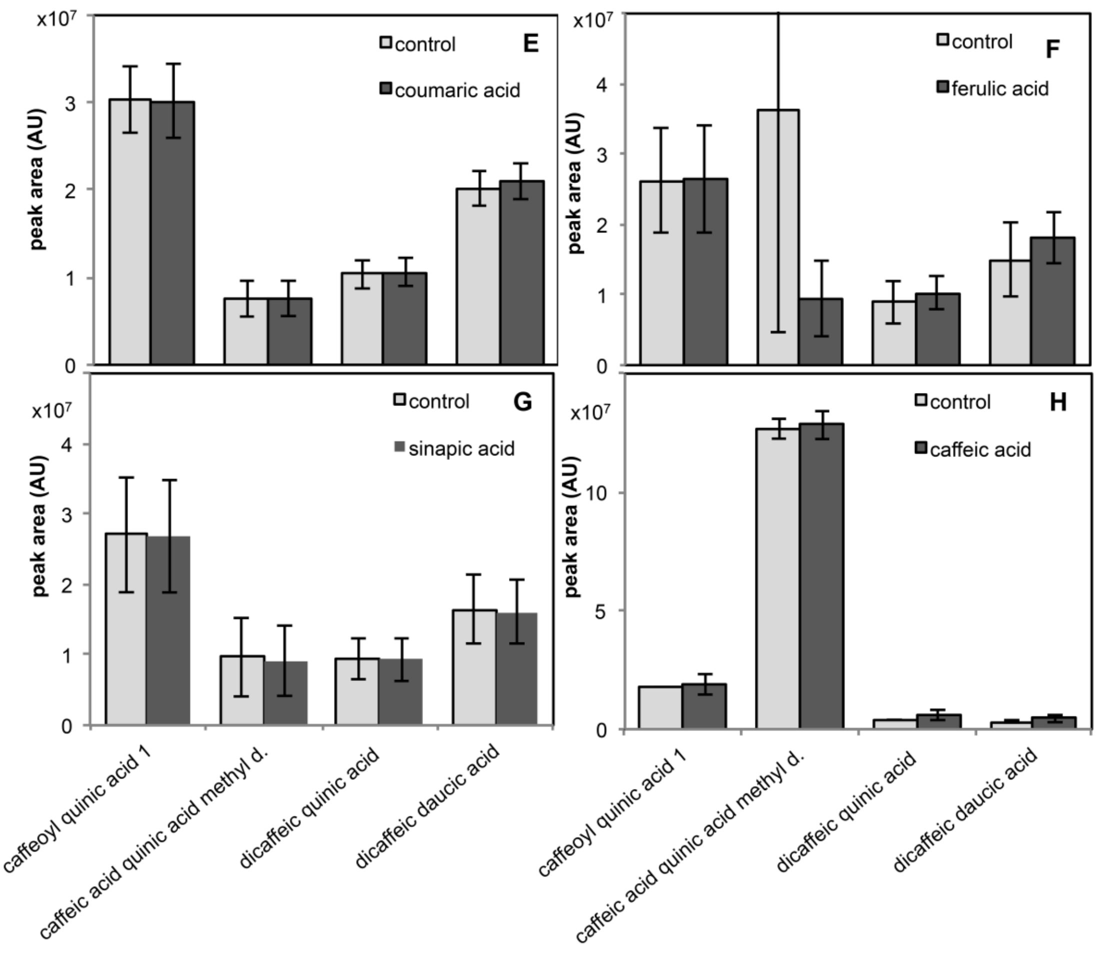

Figure S4: HPLC-ESI-MS peak areas of specific molecules or groups of molecules in R3M cell cultures fed with HCAs. Samples fed with 0.4 mM *p*-coumaric acid (A), ferulic acid (B), sinapic acid (C) and caffeic acid (D). The most consistent metabolome modification occurred in samples treated with caffeic acid (D). Interestingly, the most abundant molecules in this carrot cell line did not change with the different feeding regimes (E-H). Error bars: standard errors.

Supplemental Figure 5

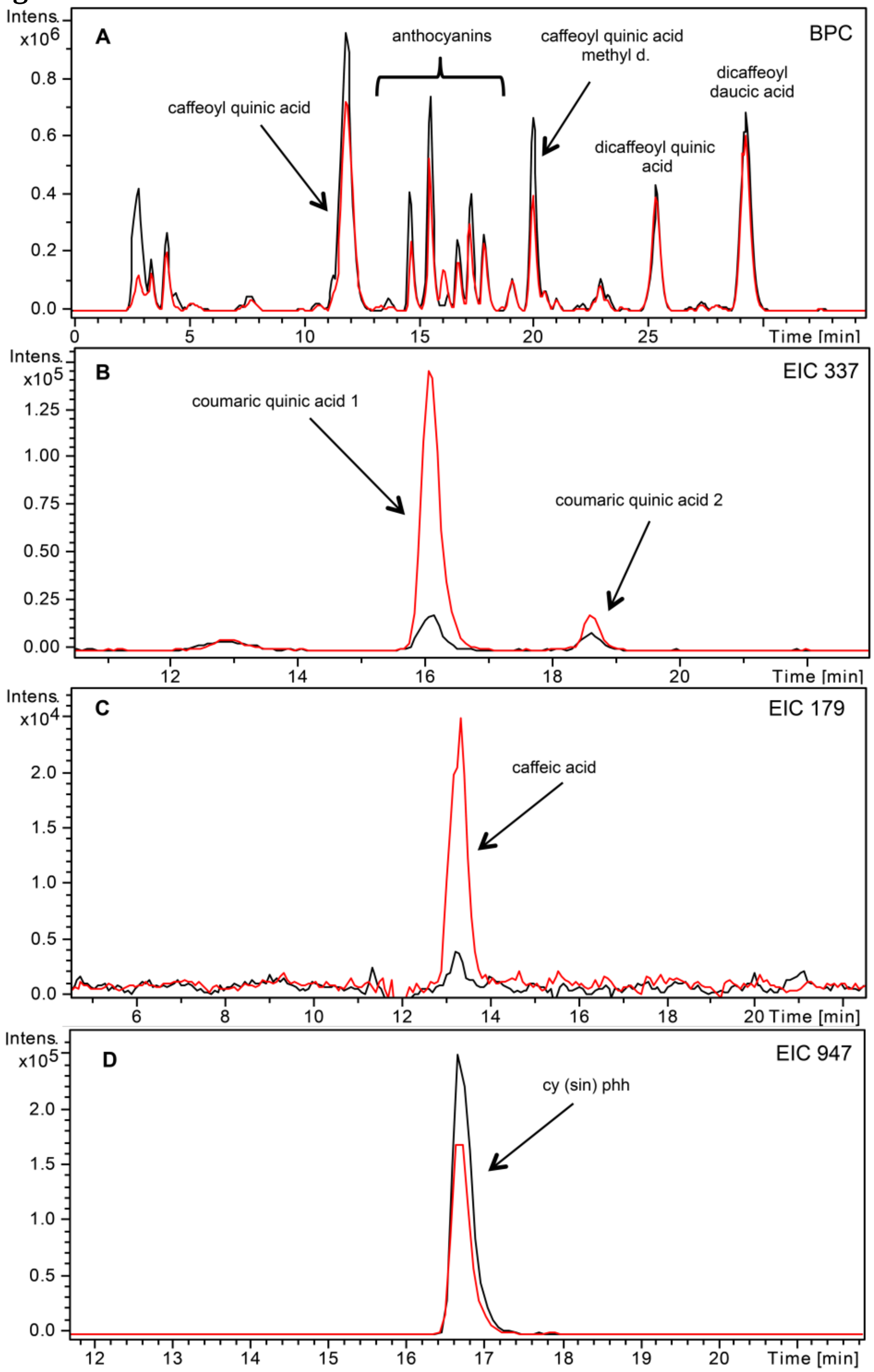

Figure S5: HPLC-ESI-MS chromatograms of R3M methanolic extracts showing trends in the abundance of heat-induced metabolites. The control and heat-stressed (1 h at 44°C) samples after 24 h recovery are represented by black and red lines, respectively. MS was carried out in negative ion mode $[M\text{-}H]^-$ and each peak shows the relative abundance of one or more detected molecules. BPC: base peak chromatogram, showing the most intense signals; EIC: extracted ion chromatogram, showing the trends of coumaric quinic acid 1 and 2 (ion 337 *m/z*), free caffeic acid (ion 179 *m/z*) and cyanidin (sinapoyl) pentose hexose hexose (ion 947 *m/z*).
